# The effect of liraglutide and sitagliptin on oxidative stress in persons with type 2 diabetes

**DOI:** 10.1038/s41598-021-90191-w

**Published:** 2021-05-19

**Authors:** Suvanjaa Sivalingam, Emil List Larsen, Daniel H. van Raalte, Marcel H. A. Muskiet, Mark M. Smits, Lennart Tonneijck, Jaap A. Joles, Bernt Johan von Scholten, Emilie Hein Zobel, Frederik Persson, Trine Henriksen, Lars Jorge Diaz, Tine W. Hansen, Henrik Enghusen Poulsen, Peter Rossing

**Affiliations:** 1 Department of Diabetes Complications Research, Steno Diabetes Center Copenhagen, Niels Steensens Vej 2, 2820 Gentofte, Denmark; 2 Department of Clinical Pharmacology, Bispebjerg Frederiksberg Hospitals, University of Copenhagen, Copenhagen, Denmark; 3 Department of Internal Medicine, Diabetes Center, Amsterdam Medical Center, Location VUMC, Amsterdam, The Netherlands; 4 Department of Nephrology and Hypertension, University Medical Center Utrecht, Utrecht, Netherlands; 5 Department of Clinical Medicine, University of Copenhagen, Copenhagen, Denmark

**Keywords:** Biomarkers, Medical research

## Abstract

Glucagon-like peptide 1 receptor agonists have shown cardioprotective effects which have been suggested to be mediated through inhibition of oxidative stress. We investigated the effect of treatment with a glucagon-like peptide 1 receptor agonist (liraglutide) on oxidative stress measured as urinary nucleic acid oxidation in persons with type 2 diabetes. Post-hoc analysis of two independent, randomised, placebo-controlled and double-blinded clinical trials. In a cross-over study where persons with type 2 diabetes and microalbuminuria (LIRALBU, n = 32) received liraglutide (1.8 mg/day) or placebo for 12 weeks in random order, separated by 4 weeks of wash-out. In a parallel-grouped study where obese persons with type 2 diabetes (SAFEGUARD, n = 56) received liraglutide (1.8 mg/day), sitagliptin (100 mg/day) or placebo for 12 weeks. Endpoints were changes in the urinary markers of DNA oxidation (8-oxo-7,8-dihydro-2′-deoxyguanosine (8-oxodG)) and RNA oxidation [8-oxo-7,8-dihydroguanosine (8-oxoGuo)]. In LIRALBU, we observed no significant differences between treatment periods in urinary excretion of 8-oxodG [0.028 (standard error (SE): 0.17] nmol/mmol creatinine, p = 0.87) or of 8-oxoGuo [0.12 (0.12) nmol/mmol creatinine, p = 0.31]. In SAFEGUARD, excretion of 8-oxodG was not changed in the liraglutide group [2.8 (− 8.51; 15.49) %, p = 0.62] but a significant decline was demonstrated in the placebo group [12.6 (− 21.3; 3.1) %, p = 0.02], resulting in a relative increase in the liraglutide group compared to placebo (0.16 nmol/mmol creatinine, SE 0.07, p = 0.02). Treatment with sitagliptin compared to placebo demonstrated no significant difference (0.07 (0.07) nmol/mmol creatinine, *p* = 0.34). Nor were any significant differences for urinary excretion of 8-oxoGuo liraglutide vs placebo [0.09 (SE: 0.07) nmol/mmol creatinine, p = 0.19] or sitagliptin vs placebo [0.07 (SE: 0.07) nmol/mmol creatinine, *p* = 0.35] observed. This post-hoc analysis could not demonstrate a beneficial effect of 12 weeks of treatment with liraglutide or sitagliptin on oxidatively generated modifications of nucleic acid in persons with type 2 diabetes.

## Introduction

Oxidative stress represents an imbalance between oxidants and antioxidants in favour of oxidants^1^, that leads to oxidative modifications of cellular components such as peptides, lipids and nucleic acids or disrupt redox signalling^2,3^. Multiple markers of oxidative stress indicate that oxidative stress is increased in persons with prediabetes or type 2 diabetes (T2D) compared to healthy controls, and has been associated with the development of micro- and macrovascular complications to T2D^4–8^. Furthermore, oxidative stress has been associated with other cardiovascular risk factors such as higher age, dyslipidaemia, smoking, hypertension and obesity^8–11^. Recently, two independent cohort studies also demonstrated that oxidative modifications of RNA, but not DNA, were associated with higher risk of cardiovascular mortality in persons with T2D^12,13^.

A recognized and validated method to assess oxidative stress is through measurement of oxidatively generated modifications of DNA and RNA as the excretion of 8-oxo-7,8-dihydro-2′-deoxyguanosine (8-oxodG) and 8-oxo-7,8-dihydroguanosine (8-oxoGuo) in the urine, respectively^14^.

Different pharmacological treatments have been suggested to influence oxidative stress^15^. Treatment with liraglutide, an analogue of human glucagon-like peptide 1 (GLP-1), has multiple effects including reduction in blood glucose, weight, blood pressure, albuminuria and lipid levels^16^. Treatment with sitagliptin, a dipeptidyl-peptidase 4 (DPP4) inhibitor has mainly effect on blood glucose^17^. Targeting the incretin system is the mechanism of action for both GLP-1 and DPP4. Large cardiovascular outcome studies have demonstrated that treatment with GLP-1 receptor agonists can reduce the risk of cardiovascular mortality and morbidity^18^. There is no convincing evidence that points to a single mechanism that can explain this cardiovascular benefit^19^. This could be mediated by the effects of liraglutide treatment on cardiovascular risk factors described above, while it has also been suggested that liraglutide treatment prevents the progression of chronic inflammation by a reduction in oxidative stress^20^.

Recently, it has been demonstrated that treatment with liraglutide reduced lipid peroxidation compared to treatment with metformin in persons with T2D^21^. Treatment with teneligliptin, a DPP4 inhibitor, has also been demonstrated to reduce urinary DNA oxidation^22^.

The aim of this post-hoc analysis was primarily to evaluate the effect of treatment with a GLP-1 receptor agonist on oxidative DNA and RNA modifications assessed as the urinary excretion of 8-oxodG and 8-oxoGuo, secondary the effect of treatment with a DPP4 inhibitor was evaluated. The analysis was conducted independently in two randomised clinical trials 1) LIRALBU (The effect of liraglutide on renal function: A randomised clinical trial); and 2) SAFEGUARD (Safety Evaluation of Adverse Reactions in Diabetes). These studies included persons with T2D and albuminuria or with T2D and obesity, respectively. We hypothesised that treatment with liraglutide reduces oxidative stress and thus the urinary excretion of 8-oxodG and 8-oxoGuo, and that this may explain some of the cardioprotective effects.

## Methods

### LIRALBU

The LIRALBU trial^16^ was a randomised, placebo-controlled, double-blinded, cross-over study including persons with T2D and microalbuminuria (n = 32). The study enrolled persons with T2D (WHO criteria), a glycated haemoglobin (HbA_1c_) ≥ 48 mmol/mol (6.5%), persistent albuminuria (> 30 mg/g in at least 2 out of 3 consecutive morning urine samples) and prescribed stable renin–angiotensin–aldosterone system blocker treatment. Key exclusion criteria included clinical heart failure and estimated glomerular filtration rate (eGFR) < 30 ml/min/1,73 m^2^. The aim of the original trial was to assess the effect of liraglutide on albuminuria as a marker of kidney damage^16^. Here we report the results of a post-hoc analysis of oxidative stress measured in the urine before and after liraglutide treatment.

**Figure 1:**
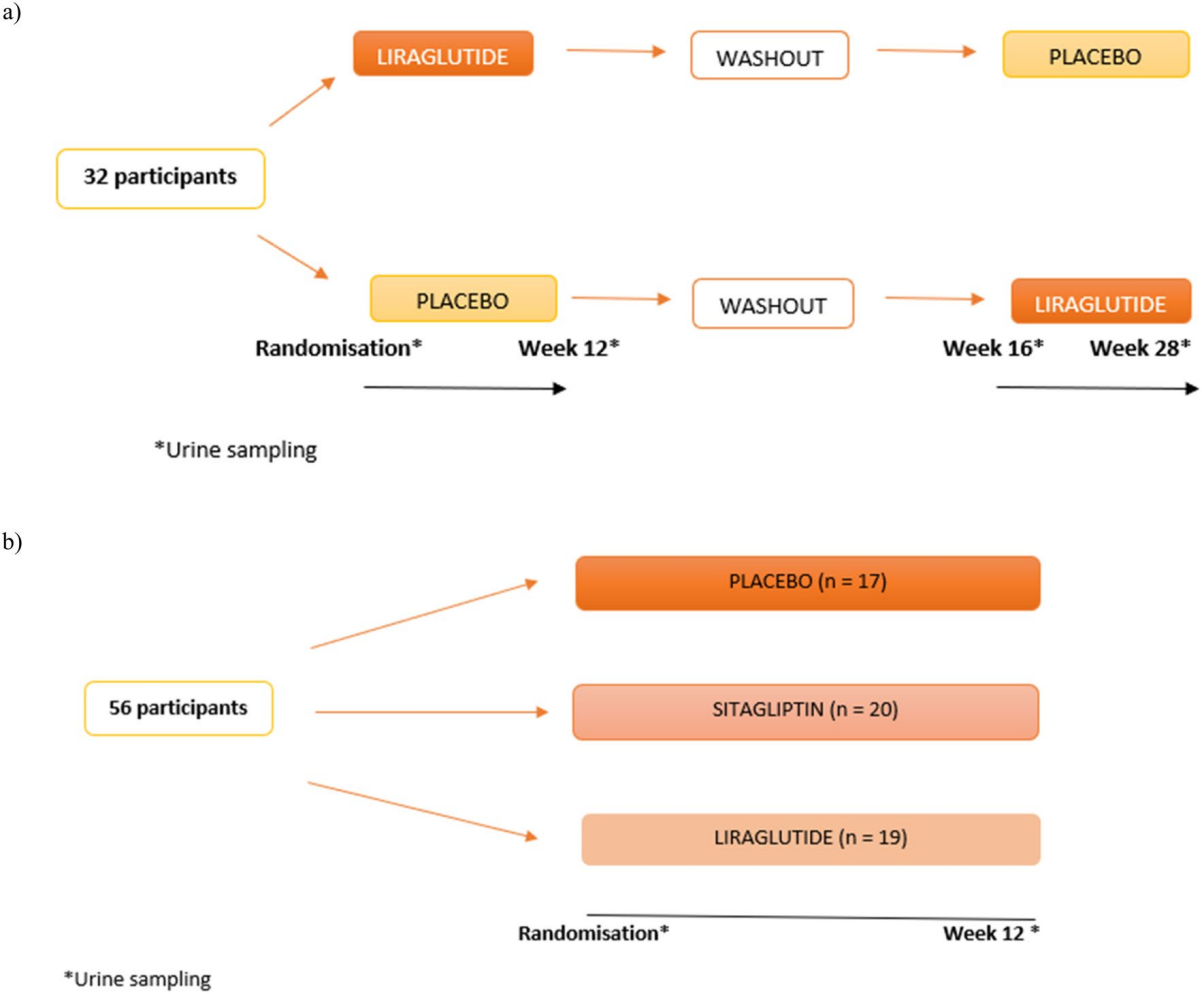
**(a)** Study design: The LIRALBU study. **(b)** Study design: The SAFEGUARD study.

Participants were randomly assigned in a 1:1 ratio to (1) 12 weeks liraglutide + standard therapy, followed by 4 weeks washout, and then 12 weeks placebo + standard therapy or (2) 12 weeks placebo + standard therapy, followed by 4 weeks washout, and then 12 weeks liraglutide + standard therapy (Fig. 1, panel a). Liraglutide/matching placebo started at 0.6 mg daily for seven days, was escalated to 1.2 mg for additional seven days, and lastly escalated to 1.8 mg daily for the remaining 10 weeks. Participants were recruited from Steno Diabetes Center Copenhagen, Denmark from April to October 2015. The study was approved by the Ethics Committee in the Capital Region of Denmark and was registered at ClinicalTrials.gov (NCT02545738).

### SAFEGUARD

The SAFEGUARD trial^23^ was a randomised, double-blinded, placebo-controlled, parallel group study including persons with T2D and obesity (n = 56). In short, participants included were men or postmenopausal women aged 35–75 years, HbA_1c_ 6.5–9.0% (48–75 mmol/mol), body mass index of 25–40 kg/m^2^ and treated with a stable dose of metformin and/or sulfonylurea for ≥ 3 months before enrolment. Key exclusion criteria included eGFR < 60 mL/min/1.73 m^2^.

The aim of the original trial was to assess the renal effect of liraglutide and sitagliptin in persons with T2D and obesity^23^.

Participants were randomly assigned (1:1:1) to 12 weeks treatment with liraglutide, sitagliptin or placebo (Fig. 1, panel b). Participants randomised to liraglutide received matching placebo prefilled pens, participants randomised to sitagliptin received matching placebo capsules and participants randomised to placebo received placebo prefilled pens and placebo capsules. Liraglutide/matching placebo started with 0.6 mg once daily during the first week, 1.2 mg once daily during the second week, and 1.8 mg once daily during the remainder of the study. Sitagliptin/matching placebo was 100 mg once daily for 12 weeks. The participants were recruited from the Diabetes Centre of the VU University Medical Center in Amsterdam, Netherlands. The study was approved by the local Ethics Review Board (2012/391) and the National Central Committee on Research Involving Human Subjects (NL41701.029.12). The study was registered at ClinicalTrials.gov (NCT01744236).

Both studies were conducted according to the Declaration of Helsinki and Good Clinical Practice. All participants gave written informed consent before any study procedure was initiated.

#### Laboratory procedures

Urine samples were stored at – 80 °C until analysis. The samples were shipped on dry-ice to the Laboratory of Clinical Pharmacology, Copenhagen, Denmark. Urinary excretion of 8-oxodG and 8-oxoGuo were determined by ultra-performance liquid chromatography tandem mass spectrometry (UPLC-MS/MS). Detailed description of the method and quality control is available elsewhere^24^. In brief, samples were heated to 37 °C and centrifuged (10,000 *g*) for 5 min, chromatographic separation by Acquity UPLC I-class system (Waters, Milford, USA) with an Acquity UPLC BEH Shield RP18 column and a VanGuard precolumn (Waters), and MS/MS detection by a Xevo TO-S triple quadrupole mass spectrometer (Waters) with a negative ionization electrospray mode^24^. Urine creatinine was determined by Jaffes method.

### Statistical analysis

The urinary excretion of 8-oxodG and 8-oxoGuo was non-normally distributed and, thus, log-transformed before all analysis.

To analyse the effect within each treatment group, we performed a paired-samples t-test to calculate the average difference with 95% confidence interval (CI), and a *P* value testing the null hypothesis that the mean difference was zero.

The SAS Enterprise Guide 7.1 was applied for all analyses and a two-sided *p* value < 0.05 was considered significant.

### LIRALBU

Differences were tested using (1) Paired samples t-test for comparisons between baseline and end-of-treatment for the liraglutide and the placebo treatment period, respectively (descriptive); and (2) linear mixed-effects models with a participant-specific random intercept to account for the correlation of repeated measurements within participants. Comparison of the change from baseline to end-of-treatment in the liraglutide treatment period vs the placebo treatment period was analysed (primary analysis). To examine the assumption of no carryover, we included a carryover-parameter in the linear model and tested for significance. Additional models included adjustment for change in weight, systolic blood pressure, and HbA_1c_ within each treatment period.

### SAFEGUARD

Differences were tested using (1) Paired samples t-test for comparisons between baseline and end-of-treatment for the liraglutide, sitagliptin and the placebo treatment groups (descriptive); and (2) ANCOVA for comparison of the change from baseline to end-of-treatment for the liraglutide vs placebo (primary analysis) group and the sitagliptin vs placebo group. Additional models included adjustment for baseline weight, systolic blood pressure, and HbaA_1c_.

## Results

### LIRALBU

A total of 32 participants were randomised, 5 participants withdrew from the study due to gastrointestinal side effects. Measurement of urinary excretion of 8-oxodG and 8-oxoGuo was not possible in 4 participants due to missing urine samples. Thus, the current analysis included 23 of the 27 participants included in the analysis of the primary endpoint (effect of liraglutide on albuminuria)^16^. The baseline characteristics of the 27 participants have been published in the primary publication^16^. No significant difference in the baseline characteristics between the participants who were randomised and who completed the study were observed. The baseline characteristics of the 23 participants included in this analysis are shown in Table 1. The majority were men (n = 19 (82.6%)), the mean (SD) age was 64.7 (7.6) years, diabetes duration was 14.3 (7.2) years, the body mass index was 32 (3.9) kg/m^2^, and the HbA_1c_ concentration was 7.8 (3.1)% (62.0 (10.2) mmol/l).

**Table 1:** Clinical characteristics at baseline.

	LIRALBU (n = 23)	SAFEGUARD (n = 47)
Age (years)	64.7 (7.6)	62.8 (6.5)
Male (%)	82.6%	78.7%
BMI (kg/m^2^)	32 (5.3)	32 (3.9)
Diabetes duration (years)	14.3 (7.2)	–
HbA_1c_ (%)	7.8 (3.1)	7.2 (0.6)
Albuminuria (mg/g)	183 (100–157)	9.6 (4.1–25.9)
Systolic blood pressure (mm Hg)	140 (17.9)	136 (15.1)
Diastolic blood pressure (mm Hg)	76 (11.1)	77 (6.7)

Data are presented as %, mean (SD) or median (IQR).

Mean relative change (CI 95%) in urinary excretion of 8-oxodG from baseline to end-of-treatment was 9.09 (− 0.92; 20.11) %, (p = 0.07) for the liraglutide treatment period and 0.23 (− 0.71; 8.14)%, (p = 0.95) for the placebo treatment period, with no difference between treatment periods either before [(0.028 (SE: 0.17) nmol/mmol creatinine, p = 0.87)] nor after (p = 0.23) adjustment.

**Figure 2:**
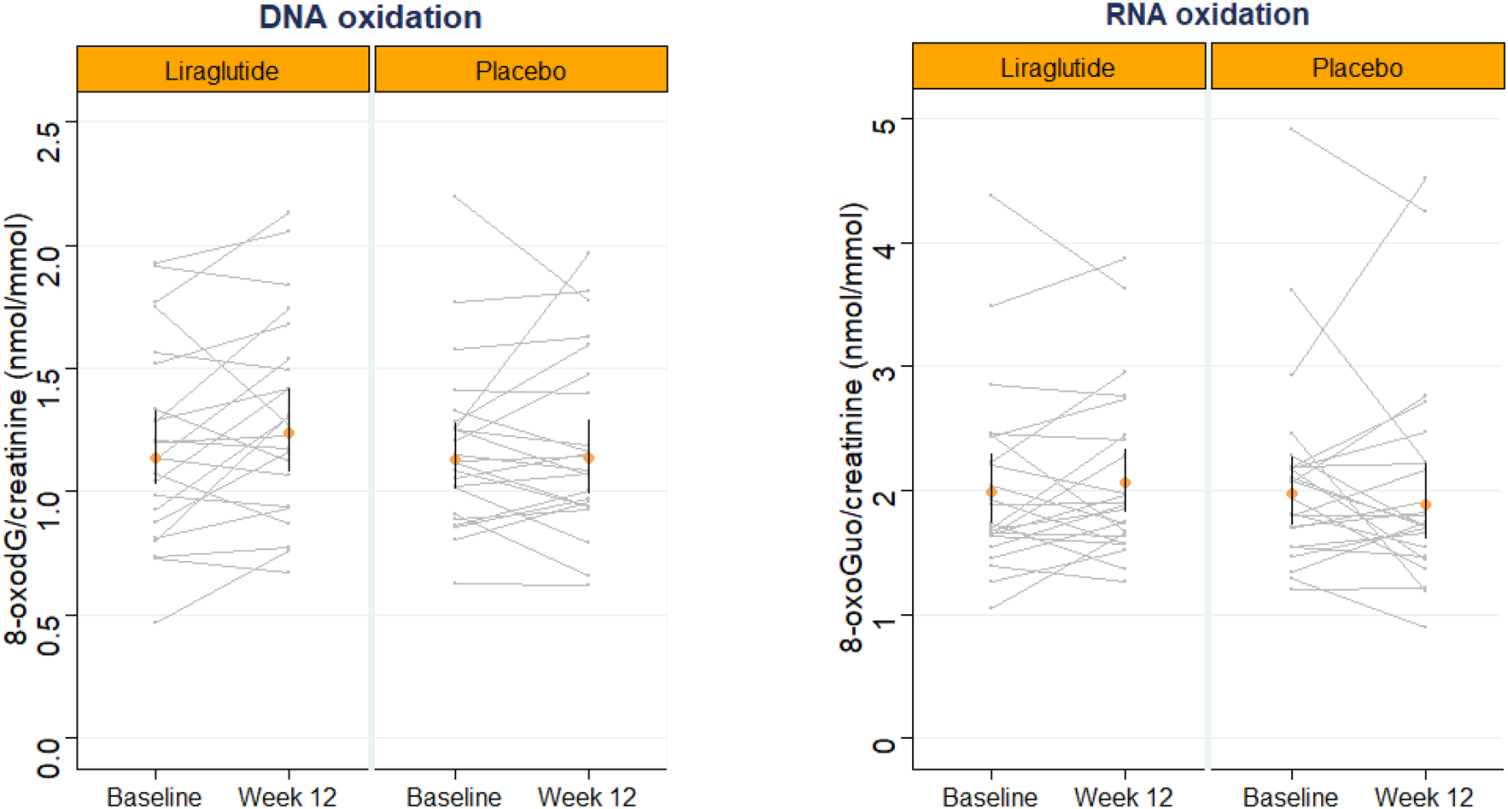
The LIRALBU study: GG-plot demonstrating individual effects after 12 weeks of liraglutide or placebo treatment in urinary excretion of 8-oxo-7,8-dihydroguanosine (8-oxoGuo) and 8-oxo-7,8-dihydro-2′-deoxyguanosine (8-oxodG). Geometric mean with 95% confidence interval limits is shown.

Mean relative change in urinary excretion of 8-oxoGuo from baseline to end-of-treatment was 2.02 (− 7.49; 12.50) %, (p = 0.68) for the liraglutide treatment period and 3.32 (− 13.52; 8.07) %, (p = 0.54) for the placebo treatment period, with no difference between treatment periods either before [(0.12 (SE: 0.12) nmol/mmol creatinine, p = 0.31)] nor after (p = 0.50) adjustment (Tables 2 and 3 and Fig. 2). No carry over effects were observed (p ≥ 0.4).

**Table 2:** Outcome measures for the LIRALBU trial.

	Placebo period			Liraglutide period			^3^*P-*value for comparison between the liraglutide and placebo period
	Baseline	End-of-treatment		Baseline	End-of-treatment		
	^1^Mean (CI 95%)	Mean (CI 95%)	% change (95% CI)	Mean (CI 95%)	Mean (CI 95%)	% change (95% CI)	
8-oxodG nmol/mmol urinary creatinine	1.13 (1.01; 1.28)	1.14 (0.99; 1.29)	0.23 (− 0.71; 8.14)	1.14 (1.03; 1.33)	1.24 (1.08; 1.42)	9.09 (− 0.92; 20.11)	0.87
			^2^p = 0.95			p = 0.07	
8-oxoGuo nmol/mmol urinary creatinine	1.98 (1.72; 2.27)	1.89 (1.61; 2.22)	− 3.32 (− 13.52; 8.07)	1.99 (1.73; 2.29)	2.07 (1.82; 2.33)	2.02 (− 7.49; 12.50)	0.31
			p = 0.54			p = 0.68	

^1^Geometric mean with 95% confidence interval.

^2^Paired-samples t-test.

^3^Unadjusted linear mixed effects.

**Table 3:** Outcome measures for the SAFEGUARD trial.

	Placebo (n = 14)			Liraglutide (n = 16)			^3^p-value	Sitagliptin (n = 17)			^4^p-value
	Baseline	EOT*		Baseline	EOT			Baseline	EOT		
	^1^Mean (CI 95%)	Mean (CI 95%)	% change (95% CI)	Mean (CI 95%)	Mean (CI 95%)	% change (95% CI)		Mean (CI 95%)	Mean (CI 95%)	% change (95% CI)	
8-oxodG nmol/mmol urinary creatinine	1.52 (1.25; 1.84)	1.34 (1.08; 1.63)	− 12.6 (− 21.1; −3.1)	1.32 (1.09; 1.62)	1.36 (1.23; 1.65)	2.8 (− 8.51; 15.49)	0.02	1.31 (1.12;1.55)	1.23 (1.03;1.46)	− 6.7 (− 14.50; 1.95)	0.34
			^2^p = 0.02			p = 0.62				p = 0.12	
8-oxoGuo nmol/mmol urinary creatinine	1.95 (1.72; 2.2)	1.88 (1.59; 2.18)	− 3.7 (− 11.85; 5.19)	1.69 (1.43; 1.99)	1.79 (1.58; 2.01)	5.9 (− 4.72; 17.64)	0.19	1.84 (1.63; 2.1)	1.91 (1.65; 2.2)	2.9 (− 8.15; 15.29*)*	0.35
			p = 0.37			p = 0.27				p = 0.60	

^1^Geometric mean with 95% confidence interval.

^2^Paired-samples t-test.

^3^Unpaired-samples t-test: change in the liraglutide treated group compared to placebo in urinary excretion of 8-oxodG and 8-oxoGuo.

^4^Unpaired-samples t-test: change in the sitagliptin treated group compared to placebo in urinary excretion of 8-oxodG and 8-oxoGuo.

*End-of-treatment.

### SAFEGUARD

A total of 56 participants were randomised and treated for 12 weeks with liraglutide (n = 19), sitagliptin (n = 20), or matching placebo (n = 17). Measurements of urinary excretion of 8-oxodG and 8-oxoGuo were missing in 9 participants (3 in each treatment group), thus the current analysis included 47 participants.

Baseline characteristics were equally distributed in the three treatment groups as previously published^25^. The baseline characteristics of the total study population is shown in Table 1. Thirty-seven (78.7%) were men, the mean (SD) age was 62.8 (6.5) years, the body mass index was 32 (5.3) kg/m^2^, and the HbA_1c_ concentration was 7.2 (0.6) % (55.9 (6.6) mmol/mol) (Table 1).

Mean change in urinary excretion of 8-oxodG from baseline to end-of-treatment was 2.8 [(− 8.51; 15.49)%, p = 0.62] for the liraglutide treated group, 6.7 [(− 14.50; 1.95)%, p = 0.12] for the sitagliptin treated group and 12.6 [(2 1.3; 3.1) %, p = 0.02] for the placebo treated group, with a significant larger increase in excretion of 8-oxodG in the group treated with liraglutide compared to placebo [0.16, (SE 0.07), p = 0.02]. Treatment with sitagliptin compared to placebo demonstrated no significant difference [0.07 (SE: 0.07) nmol/mmol creatinine, p = 0.34].

**Figure 3:**
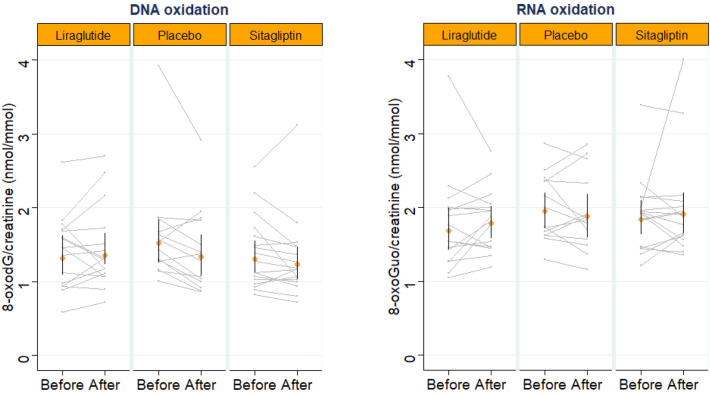
The SAFEGUARD study: GG-plot illustrating individual effects after 12 weeks of liraglutide, sitagliptin or placebo treatment in urinary excretion of 8-oxo-7,8-dihydroguanosine (8-oxoGuo) and 8-oxo-7,8-dihydro-2′-deoxyguanosine (8-oxodG). Geometric mean with 95% confidence interval limits is shown.

Mean change in urinary excretion of 8-oxoGuo from baseline to end-of-treatment was 5.9 [(− 4.72; 17.64) %, p = 0.27] for the liraglutide treated group, 2.9 [(− 8.15; 15.29*)* %, p = 0.60] for the sitagliptin treated group and 3.7 [(− 11.85; 5.19)%, p = 0.37] for the placebo treated group, with no difference between change in the liraglutide vs placebo treated groups [0.09 (SE: 0.07) nmol/mmol creatinine, p = 0.19] or change in the sitagliptin vs placebo treated groups (0.07 (SE: 0.07) nmol/mmol creatinine, p = 0.35). These results remained non-significant after adjustment (Tables 2 and 3 and Fig. 3).

## Discussion

Treatment with liraglutide reduces cardiovascular events in persons with type 2 diabetes and high cardiovascular risk, but the mechanism behind this cardiovascular benefit is not fully understood. The LIRALBU study could not demonstrate a reduction in 8-oxodG or 8-oxoGuo within liraglutide treatment compared to placebo after 12 weeks treatment in participants with T2D and albuminuria. The SAFEGUARD study demonstrated a statistically significant difference in urinary excretion of 8-oxodG in the liraglutide treated group compared to the placebo treated group after 12 weeks treatment in participants with T2D and obesity. This finding is probably mainly driven by the unexpected reduction of 8-oxodG in the placebo group as the liraglutide group was unchanged.

Oxidative stress is associated with development of diabetes and diabetic complications^4^ including cardiovascular disease^26,27^. In a previous study, it was demonstrated that high urinary excretion of 8-oxoGuo is associated with cardiovascular mortality in patients with T2D^12,13^. In a randomised clinical trial including 104 subjects with T2D, the effect of rosuvastatin treatment compared to standard care on DNA oxidation was investigated. Rosuvastatin treatment decreased the urinary excretion of 8-oxodG compared to standard care^28^. Another study with 32 participants with T2D and nephropathy receiving losartan alone or losartan + imidapril for 48 weeks showed that combination therapy decreased urinary excretion of 8-oxodG more than losartan alone^29^. The enzyme-linked immunosorbent assay (ELISA) was applied in both studies.

Liraglutide has been shown to reduce oxidative stress in experimental studies of endothelial cells from human umbilical veins due to anti-inflammatory effects^30^, and this beneficial effect on oxidative stress has been suggested to contribute to the cardioprotective effect^30^.

Previous clinical studies have described conflicting results of the effects of liraglutide on different biomarkers of oxidative stress. A randomised clinical trial including 60 participants with newly diagnosed T2D investigated the effect of treatment with liraglutide or metformin on oxidative stress and endothelial function evaluated as changes in malondialdehyde and protein carbonyls^21^. Malondialdehyde and protein carbonyls are biomarkers of lipid peroxidation and protein oxidation, respectively^31^. A significant decrease in malondialdehyde and protein carbonyl concentrations were observed after treatment with liraglutide for 6 months compared to treatment with metformin only. Furthermore, the lower concentrations of malondialdehyde were associated with reduced left ventricular myocardial deformation and improved vascular function, indicating a possible mechanism for the cardioprotective effect of liraglutide^21^.

In contrast, a recent study including 92 persons with T2D randomised into a group receiving treatment with liraglutide on top of insulin and a group treated solely with insulin for 12 weeks could not demonstrate an effect on malondialdehyde concentration in the liraglutide treated group^32^.

In the SAFEFUARD study we could not demonstrate an effect of treatment with sitagliptin compared to placebo. The effect of DPP4 inhibitors on oxidative stress in persons with TD2 and chronic kidney disease has been investigated in a randomised clinical trial (n = 45) comparing the effect of treatment with teneligliptin compared to sitagliptin. Participants treated with sitagliptin were randomised to treatment for 24 weeks with either teneligliptin or sitagliptin. In the teneligliptin treatment group, a significant decrease in urinary excretion of 8-oxodG was demonstrated. No change in urinary excretion of 8-oxodG was observed in the group treated with sitagliptin^22^. However, another study including 30 persons with T2D could not demonstrate any significant change in urinary excretion of 8-oxodG in participants treated with linagliptin, another DPP4 inhibitor^33^. This suggests that the possible effect of DPP4 inhibitors on oxidative stress might be different depending on the type of medication but larger studies are needed to confirm this^22^.

Cardiovascular outcome trials with DPP4 inhibitors has so far demonstrated safety but no cardiovascular benefit, in contrast to several GLP-1 receptor agonist studies demonstrating reduction in major adverse cardiovascular events, including the liraglutide study LEADER^18^.

Our studies, LIRALBU and SAFEGUARD, consistently failed to demonstrate a reduction in oxidative stress, assessed as DNA and RNA oxidation measured by liquid chromatography coupled with mass spectrometry, a method which previously has been used to identify subjects with high risk of cardiovascular mortality^12^. This would suggest that reduction in oxidative stress is not the mechanism contributing to the cardiovascular benefit of GLP-1 receptor agonists. It took several years for the benefit of liraglutide on cardiovascular disease to become apparent in the LEADER study, and our studies only lasted for 12 weeks, so it cannot be excluded that the treatment period was too short to capture an effect.

The strengths of our study include 1) two independent randomised controlled trials were analysed; and 2) the application of the UPLC-MS/MS technique to measure 8-oxodG and 8-oxoGuo, which is considered the reference standard method due to the high specificity, especially when compared to the ELISA method^34^. Moreover, the measurement of oxidative stress in the urine is only minimally affected by diet and cell death, and is not influenced by long-term storage and the risk of sample oxidation during collection and storage is therefore low^14^.

The limitations of the study include that the endpoints were analysed post-hoc and that the studies were not powered to detect differences in these markers of oxidative stress. The limited sample size may induce uncertainties in the analysis and larger studies are therefore needed. Due to differences in design the data from the two studies could not be combined in one analysis, but results were similar. Moreover, the majority of the participants in the LIRALBU study (82.6%) and the SAFEGUARD study were men (78.7%) and as females prior to menopause appear to have lower levels of oxidative stress than men^35^ there could be sex differences in the responses to liraglutide on oxidative stress, which may have impacted our results. However, as we are measuring changes from baseline within individuals, we assume that the impact on our findings is limited.

In conclusion, this post-hoc analysis could not demonstrate a beneficial effect of 12 weeks treatment with liraglutide or sitagliptin on oxidatively generated nucleic acid modifications in persons with T2D. Thus, this study does not support the hypothesis that the beneficial cardiovascular effect of liraglutide is partly mediated through reduction in oxidative stress.

## Abbreviations

8-oxodG: 8-Oxo-7,8-dihydro-2′-deoxyguanosine
8-oxoGuo: 8-Oxo-7,8-dihydroguanosine
GLP-1: Glucagon-like peptide 1
DPP4: Dipeptyl-peptidase 4
eGFR: Estimated glomerular filtration rate
SD: Standard deviation
SE: Standard error
T2D: Type 2 diabetes

## References

[1] Sies, H. (Sies, H. ed.) 1–8 (Academic Press, 1985).

[2] Sies, H. Oxidative stress: Concept and some practical aspects. *Antioxidants (Basel, Switzerland)* **9**, 10.3390/antiox9090852 (2020). 10.3390/antiox9090852 PMC7555448 32927924

[3] Candido, R., Srivastava, P., Cooper, M. E. & Burrell, L. M. Diabetes mellitus: A cardiovascular disease. *Curr. Opin. Invest. Drugs (London, England : 2000)* **4**, 1088–1094 (2003). 14582453

[4] West IC (2000). Radicals and oxidative stress in diabetes. Diabetic Med..

[5] Bigagli E, Lodovici M (2019). Circulating oxidative stress biomarkers in clinical studies on type 2 diabetes and its complications. Oxid. Med. Cell. Longev..

[6] Kant M (2016). Elevated urinary levels of 8-oxo-2'-deoxyguanosine, (5'R)- and (5'S)-8,5'-cyclo-2'-deoxyadenosines, and 8-iso-prostaglandin F2alpha as potential biomarkers of oxidative stress in patients with prediabetes. DNA Repair.

[7] Chou ST, Tseng ST (2017). Oxidative stress markers in type 2 diabetes patients with diabetic nephropathy. Clin. Exp. Nephrol..

[8] Thomas, M. C. *et al.* Relationship between plasma 8-OH-deoxyguanosine and cardiovascular disease and survival in type 2 diabetes mellitus: Results from the ADVANCE trial. *J. Am. Heart Assoc.* **7**, 10.1161/jaha.117.008226 (2018). 10.1161/JAHA.117.008226 PMC6064915 29960985

[9] Manna P, Jain SK (2015). Obesity, oxidative stress, adipose tissue dysfunction, and the associated health risks: Causes and therapeutic strategies. Metab. Syndr. Relat. Disord..

[10] Forstermann U, Xia N, Li H (2017). Roles of vascular oxidative stress and nitric oxide in the pathogenesis of atherosclerosis. Circ. Res..

[11] Liguori I (2018). Oxidative stress, aging, and diseases. Clin. Interv. Aging.

[12] Kjær LK (2017). Cardiovascular and all-cause mortality risk associated with urinary excretion of 8-oxoGuo, a biomarker for RNA oxidation, in patients with type 2 diabetes: A prospective cohort study. Diabetes Care.

[13] Broedbaek K (2017). Urinary albumin and 8-oxo-7,8-dihydroguanosine as markers of mortality and cardiovascular disease during 19 years after diagnosis of type 2 diabetes—A comparative study of two markers to identify high risk patients. Redox Biol..

[14] Il'yasova, D., Scarbrough, P. & Spasojevic, I. Urinary biomarkers of oxidative status. *Clin. Chim. Acta* **413**, 1446–1453, 10.1016/j.cca.2012.06.012 (2012). 10.1016/j.cca.2012.06.012 PMC3424057 22683781

[15] Kofoed Kjær, L. *et al.* Urinary nucleic acid oxidation product levels show differential associations with pharmacological treatment in patients with type 2 diabetes. *Free Radic. Res. ***53**, 694–703, 10.1080/10715762.2019.1622011 (2019). 10.1080/10715762.2019.1622011 31161826

[16] von Scholten BJ (2017). The effect of liraglutide on renal function: A randomized clinical trial. Diabetes Obes. Metab..

[17] Lo, C. *et al.* Insulin and glucose-lowering agents for treating people with diabetes and chronic kidney disease. *Cochrane Database Syst. Rev.* **9**, Cd011798, 10.1002/14651858.CD011798.pub2 (2018). 10.1002/14651858.CD011798.pub2 PMC6513625 30246878

[18] Marso SP (2016). Liraglutide and cardiovascular outcomes in type 2 diabetes. N. Engl. J. Med..

[19] Buse JB (2020). Cardiovascular risk reduction with liraglutide: An exploratory mediation analysis of the LEADER trial. Diabetes Care.

[20] Petersen KE, Rakipovski G, Raun K, Lykkesfeldt J (2016). Does glucagon-like peptide-1 ameliorate oxidative stress in diabetes? Evidence based on experimental and clinical studies. Curr. Diabetes Rev..

[21] Lambadiari V (2018). Effects of 6-month treatment with the glucagon like peptide-1 analogue liraglutide on arterial stiffness, left ventricular myocardial deformation and oxidative stress in subjects with newly diagnosed type 2 diabetes. Cardiovasc. Diabetol..

[22] Sagara M (2016). Impact of teneligliptin on oxidative stress and endothelial function in type 2 diabetes patients with chronic kidney disease: A case-control study. Cardiovasc. Diabetol..

[23] Smits MM (2015). Cardiovascular, renal and gastrointestinal effects of incretin-based therapies: An acute and 12-week randomised, double-blind, placebo-controlled, mechanistic intervention trial in type 2 diabetes. BMJ Open.

[24] Rasmussen ST (2016). Simvastatin and oxidative stress in humans: A randomized, double-blinded, placebo-controlled clinical trial. Redox Biol..

[25] Tonneijck L (2016). Renal effects of DPP-4 inhibitor sitagliptin or GLP-1 receptor agonist liraglutide in overweight patients with type 2 diabetes: A 12-week, randomized, double-blind, placebo-controlled trial. Diabetes Care.

[26] Drucker DJ (2016). The cardiovascular biology of glucagon-like peptide-1. Cell Metab..

[27] Giacco F, Brownlee M (2010). Oxidative stress and diabetic complications. Circ. Res..

[28] Abe M (2011). Effects of lipid-lowering therapy with rosuvastatin on kidney function and oxidative stress in patients with diabetic nephropathy. J. Atheroscler. Thromb..

[29] Nakamura A (2013). Combination therapy with an angiotensin-converting-enzyme inhibitor and an angiotensin II receptor antagonist ameliorates microinflammation and oxidative stress in patients with diabetic nephropathy. J. Diabetes Invest..

[30] Shiraki A (2012). The glucagon-like peptide 1 analog liraglutide reduces TNF-alpha-induced oxidative stress and inflammation in endothelial cells. Atherosclerosis.

[31] Nielsen F, Mikkelsen BB, Nielsen JB, Andersen HR, Grandjean P (1997). Plasma malondialdehyde as biomarker for oxidative stress: Reference interval and effects of life-style factors. Clin. Chem..

[32] Liu X, Huang J, Li J, Mao Q, He J (2019). Effects of liraglutide combined with insulin on oxidative stress and serum MCP-1 and NF-kB levels in type 2 diabetes. J. Coll. Phys. Surg. Pak. (JCPSP).

[33] Makino H (2019). Effect of linagliptin on oxidative stress markers in patients with type 2 diabetes: A pilot study. Diabetol. Int..

[34] Larsen EL, Weimann A, Poulsen HE (2019). Interventions targeted at oxidatively generated modifications of nucleic acids focused on urine and plasma markers. Free Radic. Biol. Med..

[35] Kander MC, Cui Y, Liu Z (2017). Gender difference in oxidative stress: a new look at the mechanisms for cardiovascular diseases. J. Cell Mol. Med..

